# Cases of Neuralgia Successfully Treated with Carbonate of Iron

**Published:** 1823-10

**Authors:** W. T. Iliff


ART. II.—*Cases of Neuralgia successfully treated with Carbonate of Iron.* By W. T. ILIFF, Esq.

ANN GUYMER, ætat. thirty-eight, and in the eighth month of her pregnancy, became a patient to the South London Dispensary, on the 10th of January last. She most heavily complained that, about ten days past, she was attacked with very severe lancinating pains over the right orbit; the paroxysms commencing about four o'clock A.M. and continuing their excruciating torments, without any intermission, until about the same hour P.M. Her general health was good, and her bowels regular. Under the impression that her sufferings might, to a certain extent, be referrible to her state of pregnancy, well-founded hopes of her recovery previous to her confinement were but faintly entertained; but, as the subcarbonate of iron had been strongly and ably recommended by Mr. Hutchinson, and the validity of that recommendation had been so satisfactorily confirmed by the concurring testimony of our most enlightened and respectable brethren, I felt not the least hesitation in prescribing it as follows:

January 11th.—℞. Ferri Subcarbon. ℈j. ter die sumend.

℞. Pil. Sapon. cum Opio, gr. v. bis sumend.

14th.—My patient thinks the pain rather decreased in violence; sleeps better. The medicines to be continued.

18th.—Has been considerably better until this morning, when she thinks that her pain had increased. An aperient powder was prescribed, and the other medicines to be repeated.

19th.—Has suffered very little since yesterday; and on the 30th was discharged perfectly free from pain.

About a month after this time she had a relapse, but the paroxysms were not so regular, either in their accession or termination. She was ordered half a drachm of the ferri subcarbonas three times a-day, and the opium to be omitted, in order that the full effect of the iron might be duly exercised. A very few days' use of it produced a considerable mitigation of her sufferings, and after a week she was discharged perfectly cured. My patient was seen several months after her discharge from the books of the dispensary, and not the slightest symptom of her previous disease existed.

Thomas Bryant, ætat. fifty-five, was admitted a patient to the South London Dispensary, on the 19th of May. He had been suffering for three weeks with an acute lancinating pain about the right scapula, shooting down the arm, and proceeding from the cutaneus, the muscula-cutaneus, the muscularis, the ulnaris, radialis, and articularis nerves. His torments were so severe as to render him incapable of following his accustomed occupations; his general health was good. Diaphoretics, colchicum, liquor arsenicalis, and a great variety of medicines, prescribed by the most eminent of our brethren, were resorted to; but all failed to afford the anxiously wished-for relief.

On the 2d of June, twenty-five grains of the ferri subcarbonas, and five grains of ginger, were directed to be taken three times a-day. On the 5th, he expressed himself evidently better, his pain being very essentially mitigated. The dose of the iron was increased to two scruples; and in about seventeen days after the first exhibition of this highly valuable discovery, and for which I must be allowed to acknowledge our great obligations to Mr. Hutchinson, my patient was discharged, having been completely relieved from all his distressing symptoms.

*Lambeth-road; 23d June*, 1823.

